# Underestimating microplastics? Quantification of the recovery rate of microplastic particles including sampling, sample preparation, subsampling, and detection using µ-Ramanspectroscopy

**DOI:** 10.1007/s00216-022-04447-z

**Published:** 2022-12-04

**Authors:** Felix Weber, Jutta Kerpen

**Affiliations:** Institute for Environmental and Process Engineering, Hochschule RheinMain, University of Applied Sciences, P.O. Box 3251, 65022 Wiesbaden, Germany

**Keywords:** Microplastic, µ-Ramanspectroscopy, Recovery rate, Sample purification, Sampling, Wastewater

## Abstract

This study is one of the first to investigate the recovery rate of high- and low-density microplastic particles (polyvinyl chloride and polypropylene) from wastewater treatment plant effluents or comparable technical facilities under nearly realistic experimental conditions. For this purpose, a method of continuous dosing of microplastic particles into an experimental flume for open-channel flow was developed. Subsequently, 12 samples were taken using volume-reduced sampling and the entire sample purification process including oxidative treatment (with hydrogen peroxide and sodium hypochlorite), density separation (with sodium polytungstate), and subsampling was carried out. Detection was conducted using automatic particle recognition and µ-Ramanspectroscopy. An average recovery rate of 27 ± 10% was determined for polypropylene microplastic particles (*d* = 53 ± 29 µm), decreasing with the particle size, and 78 ± 14% for polyvinyl chloride microplastic particles (*d* = 151 ± 37 µm). The results suggest that microplastic emissions from wastewater treatment plants are underestimated, particularly because the recovery rate of small microplastic particles < 50 µm is only 9%.

## Introduction

Microplastics (MP) in (industrial) wastewater treatment plants (WWTPs) are a highly researched topic. However, at this point, there are no standards or quality requirements for analyses. Therefore, the methods vary from study to study. To determine the analytical error, many studies present negative controls (procedural blanks) and positive controls (recovery rates). While blanks are important to control sample contamination with ubiquitous MP and to ensure the MP concentration in a sample is not overestimated, recovery rates are important to quantify false-low results. In a review of several research papers, Dimante-Deimantovica [1] showed that the recovery rate of 100-µm microplastic particles (MPPs) ranges between 40 and 80%, decreasing with the number of transfer steps in sample preparation. Other studies show that the recovery rate decreases rapidly with the particle diameter [2–5]. Unfortunately, most of the studies presenting recovery rates do not include sampling in their investigation. This creates a particularly large knowledge gap, as a sampling error can lead to serious errors in the analysis results [6].

Even though data on the recovery rate of sampling is of great interest to the scientific community [7], to the authors’ knowledge, there are few studies that present approaches to estimate recovery rates for sampling.

Bordós et al. [8] investigated the recovery rate of sampling with a cascade filtration system (50 µm and 25 µm) and µ-FTIR using spheres (100–1500 µm), fibers (1000–1500 µm), and fragments (100–300 µm) of polypropylene (PP), polyethylene terephthalate (PET), polyethylene (PE), polyamide (PA), and polyvinyl chloride (PVC). To do this, they adjusted a concentration of 100 MP/m^3^ in a stirred stainless-steel tank (2400 m^3^ of filtered tap water) and sampled 1.5 m^3^. They tested various depths of sampling and stirring modes. On average, the maximum recovery rate of all polymers was 31.4% when samples were taken from the water surface. This could be due to insufficient and non-turbulent mixing. However, sampling from a tank is not a realistic scenario for environmental or wastewater samples and the recovery rates did not include any sample preparation steps. Furthermore, the MPPs were relatively large. Therefore, this study can be used only to a limited extent to draw conclusions about sampling from aquatic environments or wastewater treatment plants. However, the data indicate that recovery rates can be low, even under controlled laboratory conditions.

Funck et al. [9] also investigated cascade filtration (100 µm, 50 µm, and 10 µm). While Bordós et al. [8] determined recovery rates for particle (MPP) concentrations (µ-FTIR), Funck et al. determined the recovery of MP mass using Py-GC–MS: 10 g of PE (*d*_50_: 120 µm, 70 µm, 10 µm) was added to 1 m^3^ of tap water in an intermediate bulk container (IBC). The total contents of the IBC were filtered, the IBC was refilled, and the contents were filtered again. The recovery rates ranged between 87 ± 2% (100 µm), 85 ± 2% (50 µm), and 88 ± 2% (10 µm). As the authors state, these values only measure the particle loss within the filtration cascade and the extraction from the filters prior to the analysis, because the IBC setup is not a realistic sampling scenario. Again, the data suggest that particle loss should be a major focus of MP analysis, as 15% of a highly concentrated sample remained in the IBC, the hoses, or the filter cascade.

Bannick et al. [10] had a similar approach: they used PE (*d*_50_: 22 µm, 52 µm, and 150 µm) and PS (*d*_50_: 298 µm) MPPs. In contrast to Funck et al. [9], the MPPs (60 mg each) were prepared for the experiment by the growth of a non-specific biofilm before they were suspended in tap water (0.15 m^3^) in stainless-steel tanks. Samples were taken using cascade filtration (100 µm, 50 µm, and 20 µm). The recovery rates ranged from 80 to 110%.

This study aims to help close the knowledge gap regarding MP sampling recovery and the following analysis protocols, especially for spectroscopic methods (particle concentrations). Spectroscopic data on MPP concentrations and size is important for ecotoxicological risk assessment [11]. For this purpose, a method of continuous dosing of MPPs in an experimental flume for open-channel flow (comparable to a WWTP effluent) was developed and recovery samples were taken under the most realistic conditions possible.

## Materials and methods

### Preparation of microplastic-suspension dosing media

#### Particle characterization

**Fig. 1:**
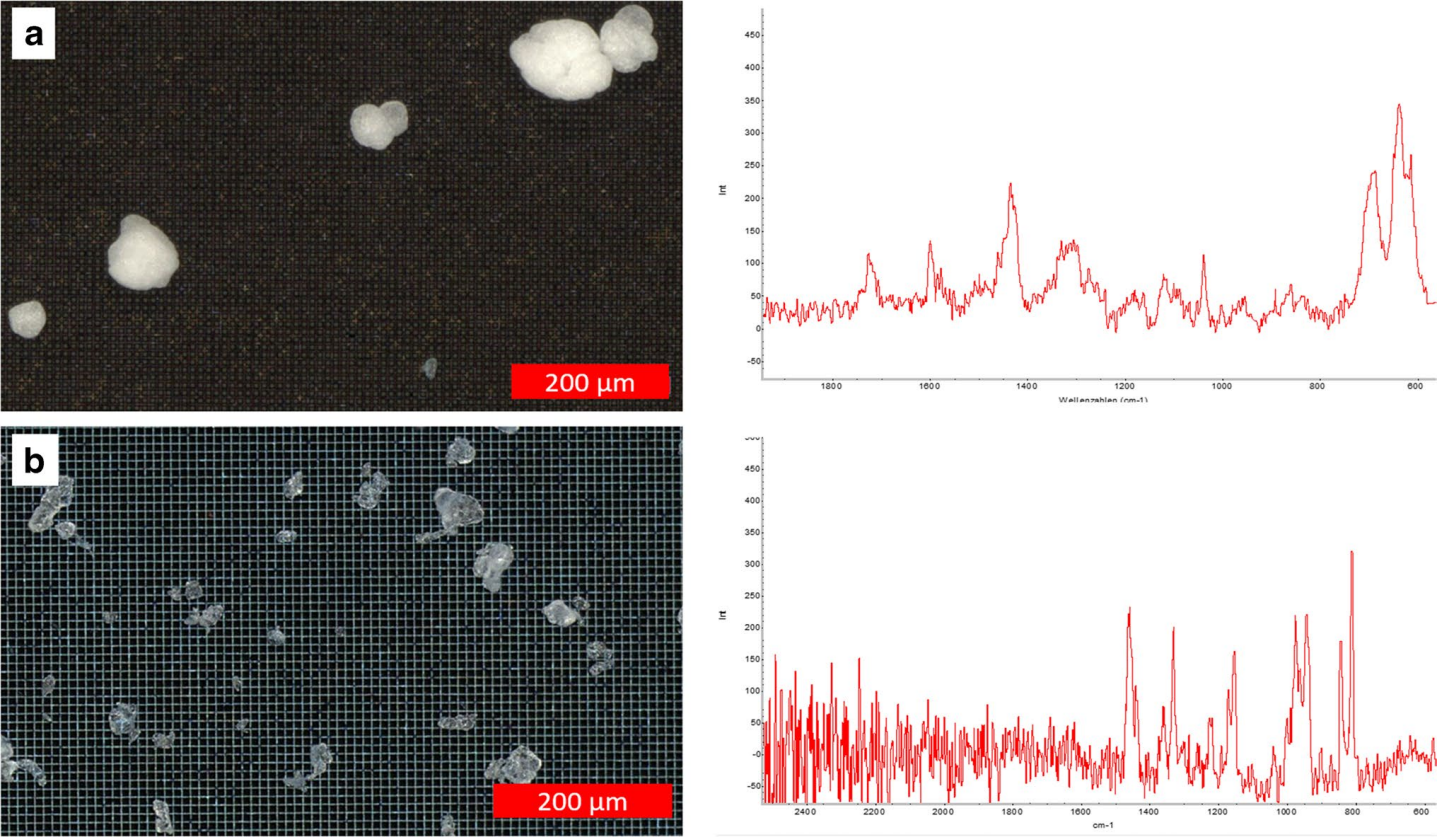
Microscopic images and spectra of PVC (**a**) and PP (**b**) MPPs

To examine differences in recovery between high- and low-density MPPs during sampling, two different types of MPPs were used: PVC (*ρ* = 1400 kg/m^3^, powder, low molecular weight, Sigma-Aldrich Co., St. Louis, USA) and PP (*ρ* = 900–910 kg/m^3^, powder, 90 µm, Goonvean Fibres Ltd., St. Austell, UK). The manufacturers’ specifications (size and morphology) for both particle types were verified: because the PVC-MPPs have a large diameter and are homogenously shaped, it was sufficient to determine their size distribution (*d* = 151 ± 37 µm) by using digital microscopy (VHX-7000, Keyence Corporation, Osaka, Japan). The Raman spectra of randomly selected PVC-MPPs were recorded. Preliminary microscopy of the PP-MPPs showed that they are very heterogeneously shaped and have a different size distribution than declared. To ensure that there was no falsification of the size distribution with non-PP-particles, grab samples (*n* = 3) of the PP-MPPs were transferred onto silicon analysis filters (Si filters, 10 µm, Smartmembranes GmbH, Halle, Germany) and analyzed using µ-Ramanspectroscopy (incl. size measurement, see section “µ-Ramanspectroscopy”). The particle size was 53 ± 29 µm (see Fig. 1).

#### Homogeneity of the microplastic suspension

To dose MPPs continuously into a stream, it was necessary to develop suitable dosing suspension media. The aim was to ensure that the MPPs neither sedimented nor floated in the dosing media during the sampling experiment: different media and mixtures were tested for PP and PVC. Since the suspension medium for PP requires a density of 900 kg/m^3^, the solutions were based on 2-propanol (*ρ* = 785 kg/m^3^, Merck KGaA, Darmstadt, Germany). To achieve a density of 900 kg/m^3^ and better particle dispersion, the alcohol was mixed with tween® 80 (polyoxyethylene (20) sorbitan monooleate, *ρ* = 1,060 kg/m^3^, VWR International LLC, Radnor, PA, USA) [12]. For PVC mixtures of saccharose (Feinzucker, Südzucker AG, Mannheim, Germany) solutions [13] and tween®80 were tested. To control the stability of the suspensions, an undefined large amount of MPPs was added, stirred, and shaken to suspend the particles homogeneously. Then the suspension was transferred to a cuvette (glass, 50 mm) immediately. To measure the stability of the suspension, the extinction of the suspension was measured for 30 min at *λ* = 860 nm according to DIN EN ISO 7027–1 [14] (Photometer DR5000, Hach-Lange, Loveland, USA).

### Microplastic particle dosing

The requirements for dosing were set as follows: the dosing stream should be continuous, without fluctuations in the volume flow, and with a steady outflow (no drops). To this end, a glass syringe without a cannula (Fortuna Optima®, Poulten & Graf GmbH, Wertheim, Germany) was used for the PP, and a burette (straight valve stopcock, Hirschmann Laborgeräte GmbH & Co. KG, Eberstadt, Germany) for the PVC dosing medium. A different equipment was necessary because the two dosing media have different viscosities. While dosing, the liquid level was kept constant (PP: 15 ml, PVC: 50 ml). Volume flow was at approx. 25 ml/min (PP) and 4.7 ml/min (PVC). The actual average dosing volume flows were determined gravimetrically after the experiment. Based on a volume flow of 80 l/s (see section “Recovery experiment”) in the experimental flume (see section “Sampling”), the required concentration of MPPs in the dosing suspension was estimated. Starting from the assumption of spherical MPPs, the mass of MPPs required for a stock solution was calculated. Since the conversion of MP mass into MPP concentrations is error-prone, the concentration of the MPPs was checked using µ-Ramanspectroscopy and adjusted by dilution until the required particle concentration was achieved: 3470 ± 307 PP-MPP/ml (*n* = 3) and 9500 ± 1200 PVC-MPP/ml (*n* = 3). The concentrations were different because different dosing media with different viscosities and dosing volume flows were chosen for each polymer.

### Recovery experiment

#### Sampling

The recovery experiment was conducted at an experimental flume for open-channel flow in the hydraulic engineering laboratory at RheinMain UAS (Kurt-Schumacher-Ring 18, 65,197 Wiesbaden, Germany). The experiments with PVC and PP were conducted on different days (PVC: 09/27/2021, PP: 05/20/2022) but with identical setups and methods.

The flume had a length of 27.5 m and a width of 0.595 m and was located outside. The wall material was made of a textured coated board. The bottom was covered with artificial lawn (PE). The water used for experiments was tap water, stored in a 250 m^3^ underground basin for years. It has already been used for several experiments. Therefore, some of its parameters are different from common tap water: chemical oxygen demand (COD) = 6.1 mg/l, total suspended solids (TSS) = 1 mg/l, and pH = 8.15. From the basin, pumps circulated the water through the flume. Before the experiment began, the volume flow in the flume was adjusted and measured (81 l/s for PVC [${v}_{d}$ = 0.85 m/s] and 77 l/s [${v}_{d}$= 0.8 m/s] for PP). The water level in the flume was 0.16 m. The flow conditions were turbulent (Reynolds 3 × 10^5^). Therefore, it can be assumed that the MPPs were homogeneously distributed in the water. As the water circulated in the system, the hydraulic retention time was calculated (51–54 min) and the maximum time span for the dosing experiment was set at 40 min to ensure that no dosed particle passed the flume twice. With a volume flow of about 290 m^3^/h, the flow conditions in the experimental flume were comparable to a flume at the effluent of a final clarifier of a municipal WWTP (10,000–100,000 population equivalents) or an industrial WWTP.

The dosing system was installed 2 m away from the inlet of the flume. The outlets of the syringe and the burette were placed as closely as possible to the water surface. A stirrer (diameter: 135 mm, 166 rpm) was placed below the water surface to ensure that the MPPs were properly suspended. The sampling system was installed 17 m away from the outlet of the flume (15 m away from the dosing point).

**Fig. 2:**
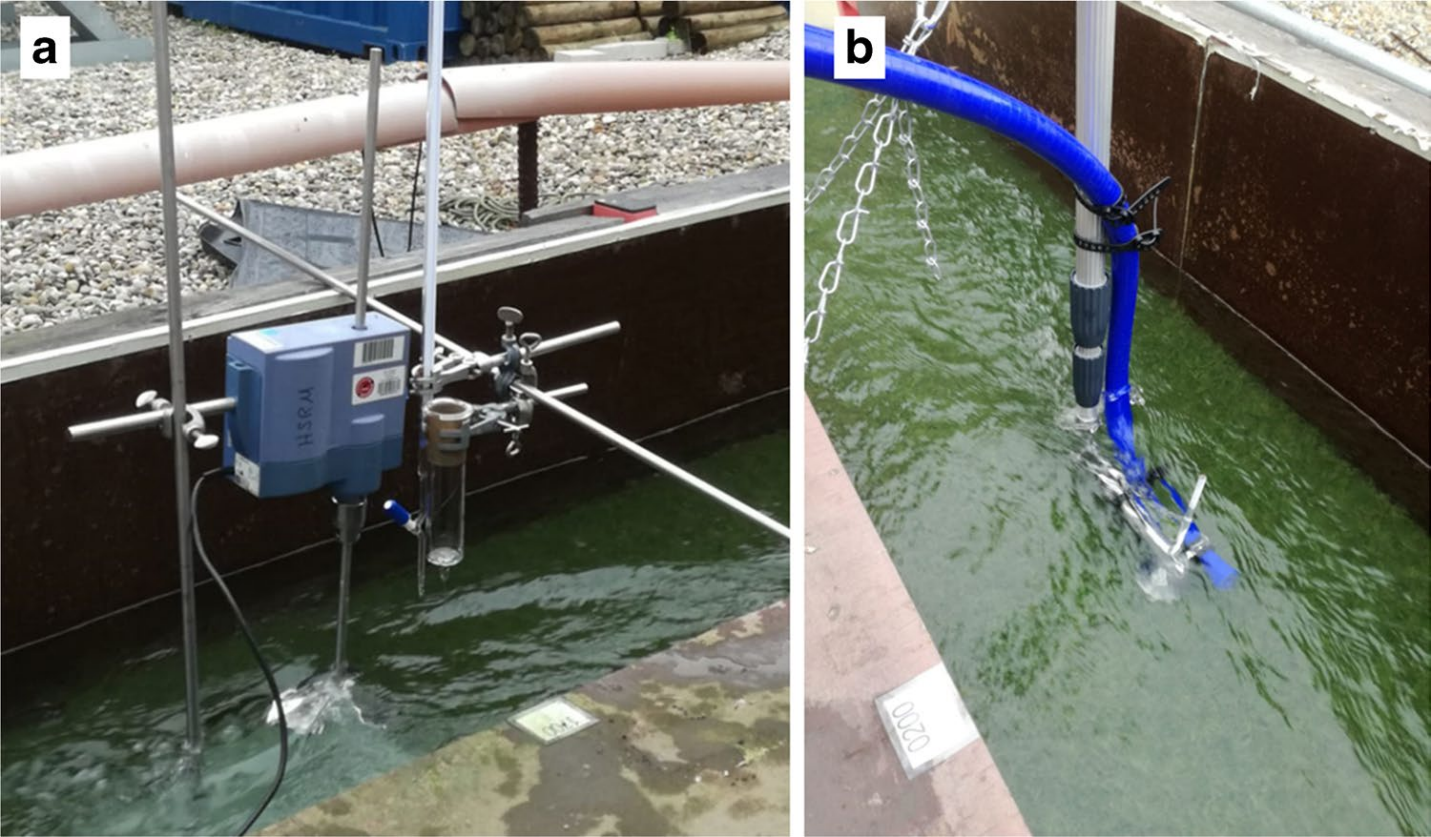
MPP dosing (**a**) and sampling at the experimental flume (**b**)

For sampling, water was drawn through stainless-steel cartridge filters (suction side, 10 µm, acuraScreen, Fuhr GmbH, Klein-Winternheim, Germany) by using a centrifugal pump (VGX 9/10, SPECK Pumpen Verkaufsgesellschaft GmbH, Germany). To ensure identical sampling conditions throughout the experiment, the sampling hose (silicone, inner diameter 1.905 cm) inlet was placed in the middle of the flume and at mid-water level in accordance with DIN 38402–11 [15] and DIN 38402–24 [16] (see Fig. 2).

To verify whether the sampling was reproducible, six samples (six different filters) were taken for 5 min and with a pause between sampling periods of 1 min each. The sample volume ranged from 125 to 159 l.

The sampling volume flow decreased during each sampling period from 35 to 25 l/min (${v}_{n}$ =1.76 m/s on average), caused by clogging of the filters. Thus, isokinetic sampling conditions were not present ($\frac{{v}_{n}}{{v}_{d}}>2, \text {on average}$).

#### Blanks

Measures of contamination mitigation were conducted as in Wolff et al. [17].

To determine the PVC and PP background concentration in the flume, 133-l and 162-l samples (“flume blanks”) were taken from the flume before MPPs were dosed, respectively. The blanks were treated in the same way as the recovery samples (see section “Sample preparation”). In parallel, procedural blanks for the sample preparation procedure were determined (*n* = 7) as in Wolff et al. [17]. Based on the procedural blanks, the limit of quantification (LOQ) was determined in accordance with DIN 32645 [18] (see Eq. 1) for both PP and PVC:

1 $$LOQ={\overline{x} }_{blank}+10*{SD}_{blank}$$

where $${\overline{\mathrm{x}} }_{\mathrm{blank}}\text{ is the arithmetic average of the blank values}$$  

and $${\mathrm{SD}}_{\mathrm{blank}}\text{ is the standard deviation of the blank values}$$  

#### Sample preparation

The sample preparation was conducted in a manner similar to Wolff et al. [17], but with a different density separation.

To eliminate biological organic matter in the sample matrix, an oxidative treatment was conducted. The samples were treated with hydrogen peroxide (H_2_O_2_) (p.a. 50%, Carl Roth GmbH & Co. KG, Karlsruhe, Germany) for 24 h at 323.15 K. In a second step, the samples were transferred onto a stainless-steel filter membrane and treated with 0.3 l of sodium hypochlorite (NaClO) (12% tech., Carl Roth GmbH & Co. KG, Karlsruhe, Germany) at room temperature for 6 days.

To reduce particle losses during transfer steps, all materials with sample contact (e.g., beakers, tweezers) were rinsed with n-hexane (HiPerSolv CHROMANORM, VWR, Radnor, PA, USA) from a syringe and pure water from a squirt bottle made of perfluoroalkoxy alkanes (PFA).

Similay to the method applied by Wolff et al. [18], who used ZnCl_2_ as a density separation agent, the sample was rinsed from the filter membrane into a sodium polytungstate solution (SPT) (*ρ* = 1.7 g·ml^−1^, Carl Roth GmbH & Co. KG, Karlsruhe, Germany) with n-hexane after oxidation. n-Hexane is not mixable with SPT and the supernatant was vaporized. The SPT suspension was transferred into glass centrifugation tubes (100 ml, Sigma Laborzentrifugen GmbH, Osterode am Harz, Germany) and centrifuged for 5 min at 2400 rpm (Sigma 3–16 L, Sigma Laborzentrifugen GmbH, Osterode am Harz, Germany). Similar to the method applied by Jekel et al. [19], the centrifugation tubes were frozen at 243.15 K for a minimum of 12 h. Afterwards, the upper 3 cm of the frozen SPT was thawed and rinsed into a beaker by using pure water from a squirt bottle.

The recovery rates obtained using this new method were determined for the size classes 22–27 µm and 45–53 µm using spherical fluorescent PE-MPPs (Cospheric LLC, Santa Barbara, CA, USA) and the PVC-MPPs used for the dosing experiment as triplicate. Regardless of the particle size, the recovery rate is 95% ± 3.5% for PE and 93 ± 3% for PVC-MPPs.

Three subsamples (3%) of each sample were taken as in Wolff et al. [17] after oxidative treatment and density separation. Subsampling was necessary because of a high concentration of PE and non-plastic particles in the samples (see section “Experimental design”).

#### µ-Ramanspectroscopy

The measurements were conducted using a µ-Raman spectroscope (DXR2xi, Thermo Fisher Scientific Inc., Waltham, MA, USA) with a front-illuminated EMCCD detector. For measurements, the electron multiplier (EM) was turned off. All particles from 20 to 140 µm (PP samples) or > 50 µm (PVC samples) on the filter were detected using the automatic particle recognition feature of the instrumental software OMNICxi (v.2.3, Thermo Fisher Scientific Inc., Waltham, MA, USA). Each detected particle was analyzed with a laser wavelength of 785 nm, a laser power of 8 mW, and a total exposure time of 6.75 s (three repetitions of 2.25 s each). The objective used had a 20 × magnification and a numerical aperture of 0.45. Spectra were recorded in the range of 50–3300 cm^−1^ and with a resolution of 5 cm^−1^.

The spectra recorded were compared with the reference library P/N L60001 (S.T. Japan Europe GmbH, Köln, Germany) using OMNICxi and OMNIC (v 9.11.706, Thermo Fisher Scientific Inc., Waltham, MA, USA). Due to a large number of false positive and false negative results, all spectra were manually checked by a researcher. The particle size was determined automatically by OMNICxi based on the greatest particle diameter. Since the dosed MPPs were white or transparent, no colored MPPs were included. In addition, no MP fibers were included.

## Results

### Microplastic-suspension dosing media

**Fig. 3:**
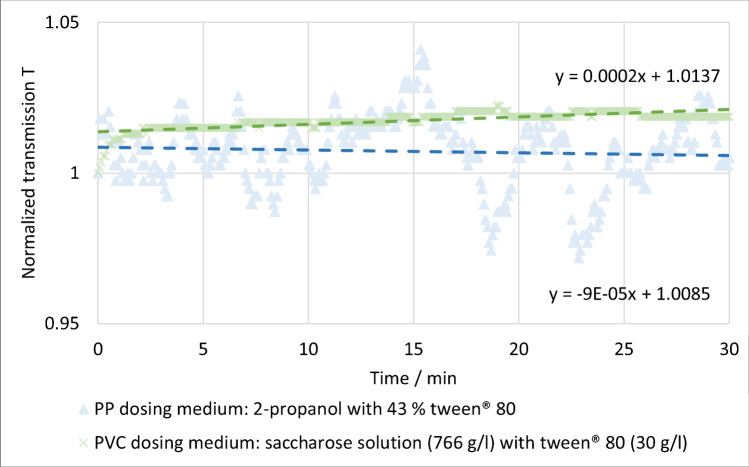
Normalized transmission of the dosing media with MPPs at *λ* = 860 nm

For PP, a mixture of 2-propanol with *w* = 43% tween® 80 yielded the best results, while PVC could be suspended homogenously in an aqueous saccharose (766 g/l) and tween® 80 (30 g/l) solution (low slope of the linear line of best fit and low fluctuations < 5% (see Fig. 3)).

### Microplastic particle dosing

The average volume flow of the PP-dosing medium was 25.2 ml/min, resulting in a target concentration of 19,200 ± 1500 PP-MPPs/m^3^ (see section “Recovery rates”).

An accumulation of air bubbles restricted the outlet of the burette during the PVC experiment, diminishing the dosing volume flow. This effect was observed during the experiment, as a result of which the dosing was subsequently simulated in the laboratory under similar conditions (*T* = 290.15 K) to determine the volume flow and the corresponding PVC-MPP concentration in the flume for each sample. The dosing volume flow went from 5.2 to 4.3 ml/min, causing a target concentration reduction from 10,110 ± 1210 PVC-MPPs/m^3^ to 8360 ± 1000 PVC-MPPs/m^3^.

The concentrations correspond to the concentrations reported at industrial and municipal WWTPs [20].

### Blanks

#### Procedural blanks

The procedural blank for PVC-MPPs > 100 µm is 0.3 ± 0.8 PVC-MPPs per analysis. This yields an LOQ of 8 PVC-MPPs per analysis. The procedural blank for PP-MPPs is 0.9 ± 0.7 PP-MPPs per analysis, also resulting in a low LOQ of 8 PP-MPPs.

#### Flume blanks

The PVC-MPPs used could be easily distinguished from other PVC-MPPs using microscopy prior to µ-Ramanspectroscopy, and no similar PVC-MPPs were identified in the flume blank. The blank value can be considered as 0 PVC-MPPs/m^3^.

The flume blank for PP-MPPs was 5100 ± 1540 PP-MPPs/m^3^ (20–50 µm: 43%, 50–100 µm: 46%, 100–140 µm: 11%). Each size class of the results of the PP recovery experiments was corrected by the corresponding blank value.

### Recovery rates

Table 1 shows the average recovery rates of all samples (PVC and PP). The PVC-MPP recovery rate is 78 ± 14%, while the PP-MPP recovery is 27 ± 10%. In both cases, the recovery rates fluctuated between the samples (average value and standard deviation (SD) of all *n* = 6 samples) and between the subsamples of one sample (SD of each sample, *n* = 3). While the SD of each sample represents the error of the subsampling procedure and the detection by means of using µ-Ramanspectroscopy, the average recovery rate shows the error of the analysis protocol as a whole (sampling + sample purification + subsampling + detection).

**Table 1:** Average recovery rate of all samples

Sample	Target concentration, MPPs/m^3^	Detected concentration, MPPs/m^3^	Average recovery rate, %
PVC 1	10,110 ± 1210	6350 ± 1380	63 ± 14
PVC 2	9450 ± 1130	6920 ± 2050	73 ± 22
PVC 3	8760 ± 1050	7000 ± 1090	80 ± 12
PVC 4	8700 ± 1040	5500 ± 240	63 ± 3
PVC 7	8520 ± 1020	7930 ± 1340	93 ± 16
PVC 6	8360 ± 1000	8050 ± 2000	96 ± 24
PVC on average			78 ± 14
PP 1	19,200 ± 1500	3810 ± 1000	20 ± 5
PP 2		6700 ± 1800	35 ± 9
PP 3		6610 ± 770	35 ± 4
PP 4		2600 ± 330	14 ± 2
PP 5		4100 ± 1200	22 ± 6
PP 6		6970 ± 420	37 ± 2
PP on average			27 ± 10

**Fig. 4:**
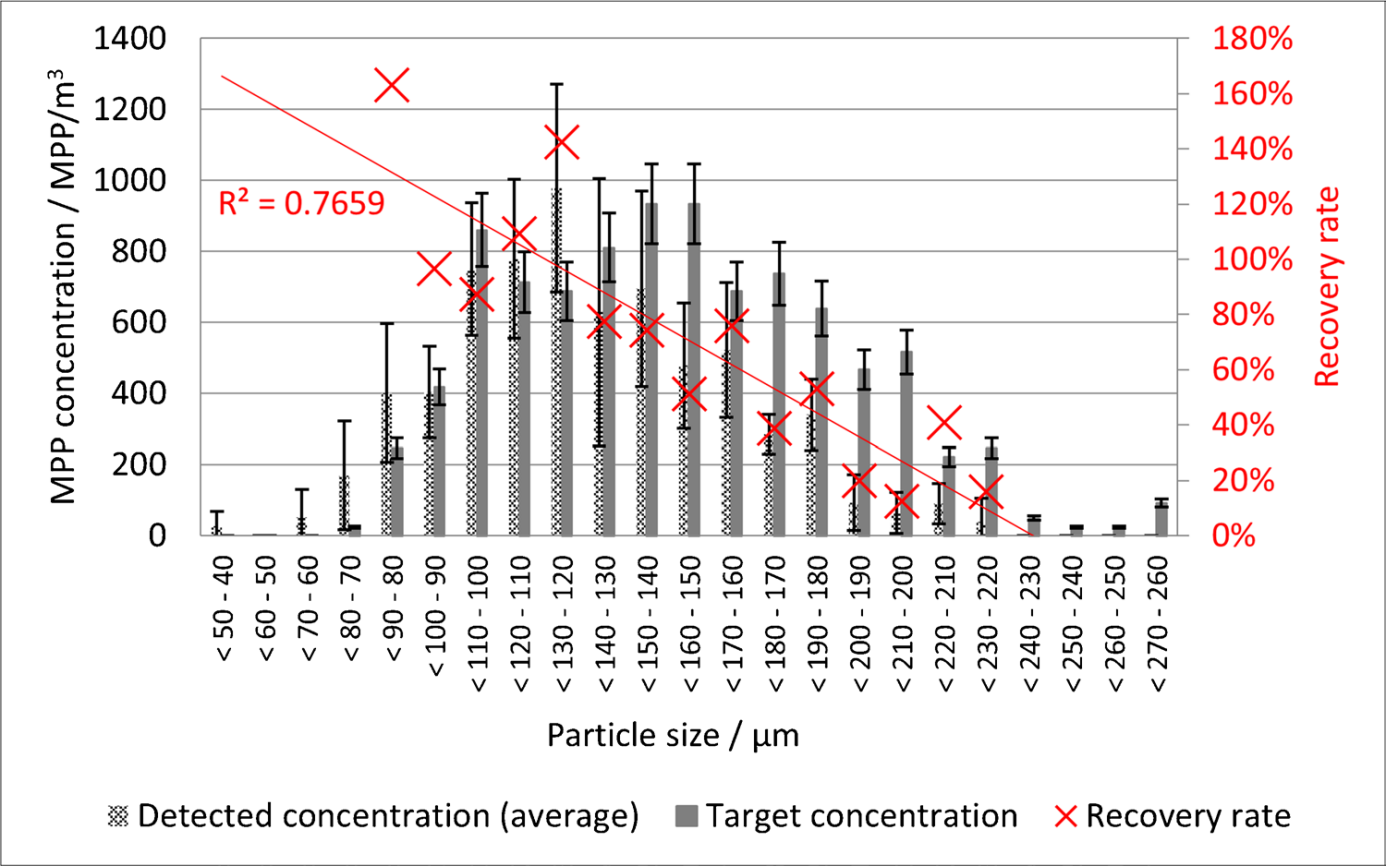
Average observed PVC-MPP concentration by size range compared to the target value and average recovery rate of the PVC-MPPs compared to particle size

The average size of the recovered PVC-MPPs changed significantly (single-factor ANOVA, *α* = 0.05, MS Excel 2016, Microsoft Corporation, Redmond, WA, USA) from 150 ± 37 µm to 131 ± 35 µm. This led to a higher recovery rate with decreasing particle size (see Fig. 4).

**Fig. 5:**
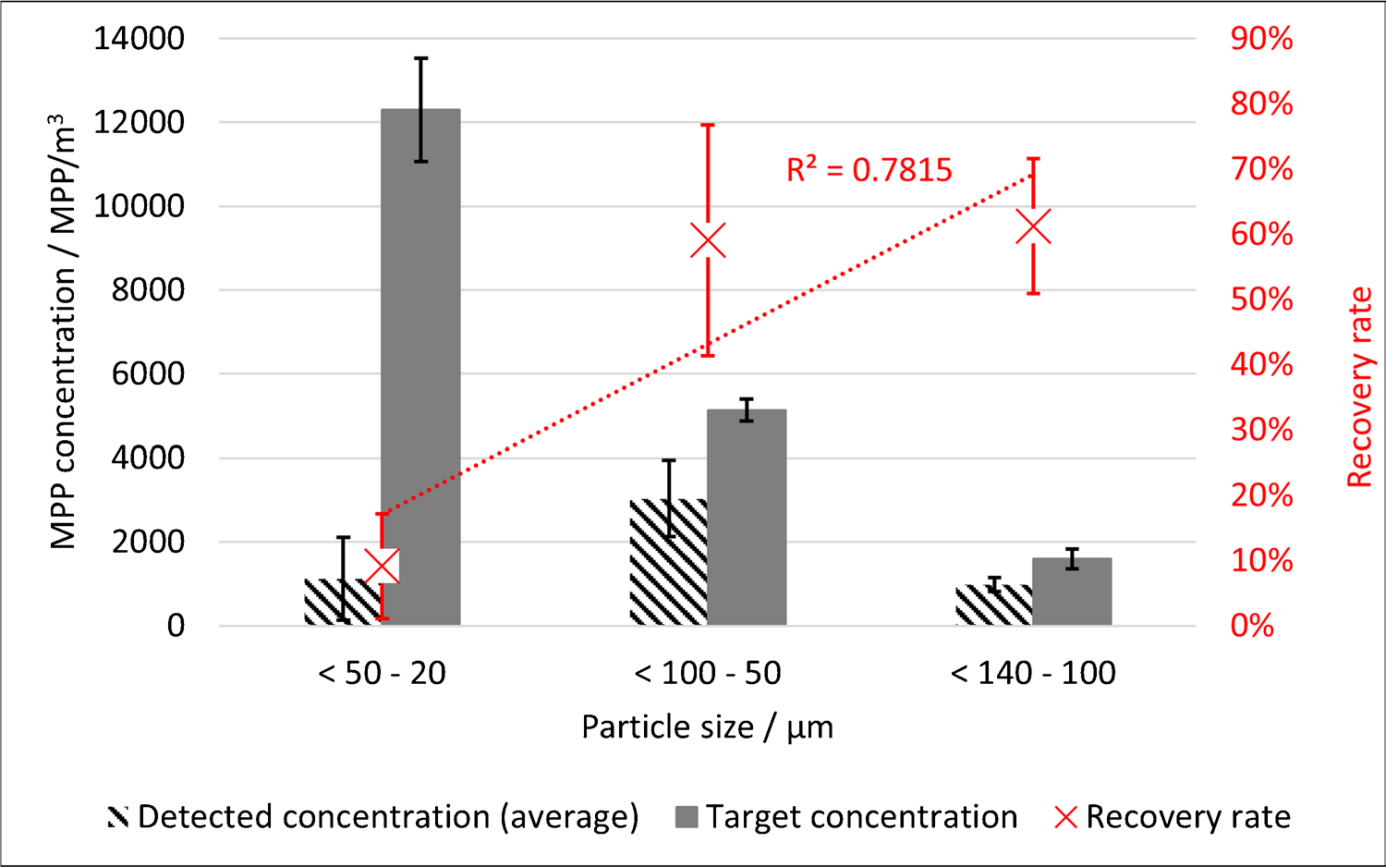
Average PP-MPP concentration by size range compared to the target value and average recovery rate of the PP-MPPs compared to particle size

Due to higher fluctuations in the particle sizes between samples and aliquots, probably caused by the heterogenous particle morphology, the size classes recommended by Braun et al. [21] were used for the PP-MPP recovery evaluation. The recovery rate declines from 61% for 100–140 µm to 9% for 20–50 µm (see Fig. 5).

## Discussion

### Experimental design

The experiment design aimed to mimic realistic conditions as closely as possible with reasonable effort. However, there are some deviations from real conditions when sampling WWTP effluents. One difference is the TSS concentration, which is below 20 mg/l in WWTP effluents [22]. The TSS concentration has a major effect on the sampling because suspended matter clogs the cartridge filters and thus changes the (iso-) kinetic ratio of the sampling. In this study, the kinetic ratio was $\frac{{v}_{n}}{{v}_{d}}>2$ on average. This may have resulted in a false-high sampling recovery: assuming that particles are homogeneously suspended in a stream, they are sucked into the sampling system representatively at a ratio of $\frac{{v}_{n}}{{v}_{d}}=1$, since no diversion occurs through increased or decreased flow velocity at the sampling hose inlet. At a ratio of $\frac{{v}_{n}}{{v}_{d}}<1$, smaller particles flow past the sampling hose inlet following the main flow due to their lower inertia and are underrepresented. In the case of a ratio of $\frac{{v}_{n}}{{v}_{d}}>1$, smaller particles are sucked into the sampling system and are overrepresented due to the same effect. This is particularly interesting in the present study, as a ratio of $\frac{{v}_{n}}{{v}_{d}}>2$ was applied. Nevertheless, the smallest size fraction shows the lowest recovery rate.

Since MP is well removed in the activated stage [20], it can be assumed that MP is bound in activated sludge flocs. Thus, it is suspected that MP in the effluent of a WWTP is also embedded in sludge flocs or at least covered with a biofilm. This may influence the dispersion of MPPs in water.

Usually, flumes in WWTP effluents are made of concrete and show a varying growth of algae on the bottom and the walls. The flume in this study was made of textured coated board and algae at the flume bottom were simulated using an artificial lawn. It can only be speculated whether MP adheres better to concrete or to coated board, and whether algae or artificial lawns have the greater MP retention capacity.

Not all sampling sites at the effluent of a WWTP are located at flumes. In some cases, the sample must be taken from the secondary clarifier effluent. Conditions similar to those in this study can be assumed here. In other cases, only pipes are available, resulting in completely different conditions.

The sampling of six replicates in short time intervals is not a common sampling strategy. As a rule, cartridge filters are used as a cascade or individually. Also, a short sampling period of 5 min (grab sample) is not recommended. In wastewater treatment, 2-h mixed samples are often used. Since it is difficult to filtrate for 2 h if the TSS concentration is high, the mixed sample can be designed alternating with fixed time intervals in accordance with DIN EN ISO 5667–1 [23]. The purpose of spreading the samples over six filters was to investigate whether there are large variations in results over a sampling period under the same conditions. The effects on the results of sampling and sample preparation could not be distinguished from each other. The results showed that there were fluctuations between the samples. They were not negligible, indicating that it is necessary to analyze multiple samples. Further, the six filters allowed us to investigate the reproducibility of the subsampling and analysis. The magnitude of the fluctuations is acceptable, especially for PVC larger than reported by Wolff et al. [17], who validated the method with PE and without other interfering MPPs or non-MP particles. Thus, it is conceivable that errors also occurred during particle recognition and detection. At least three aliquots should be examined to smooth fluctuations.

The methods applied for sampling, sample preparation, and detection could be one reason for the low PP recovery rate. However, this is unlikely, since WWTP emission data obtained using these methods are similar to MP concentrations measured by other research groups who have applied comparable methods [17, 24–27].

**Fig. 6:**
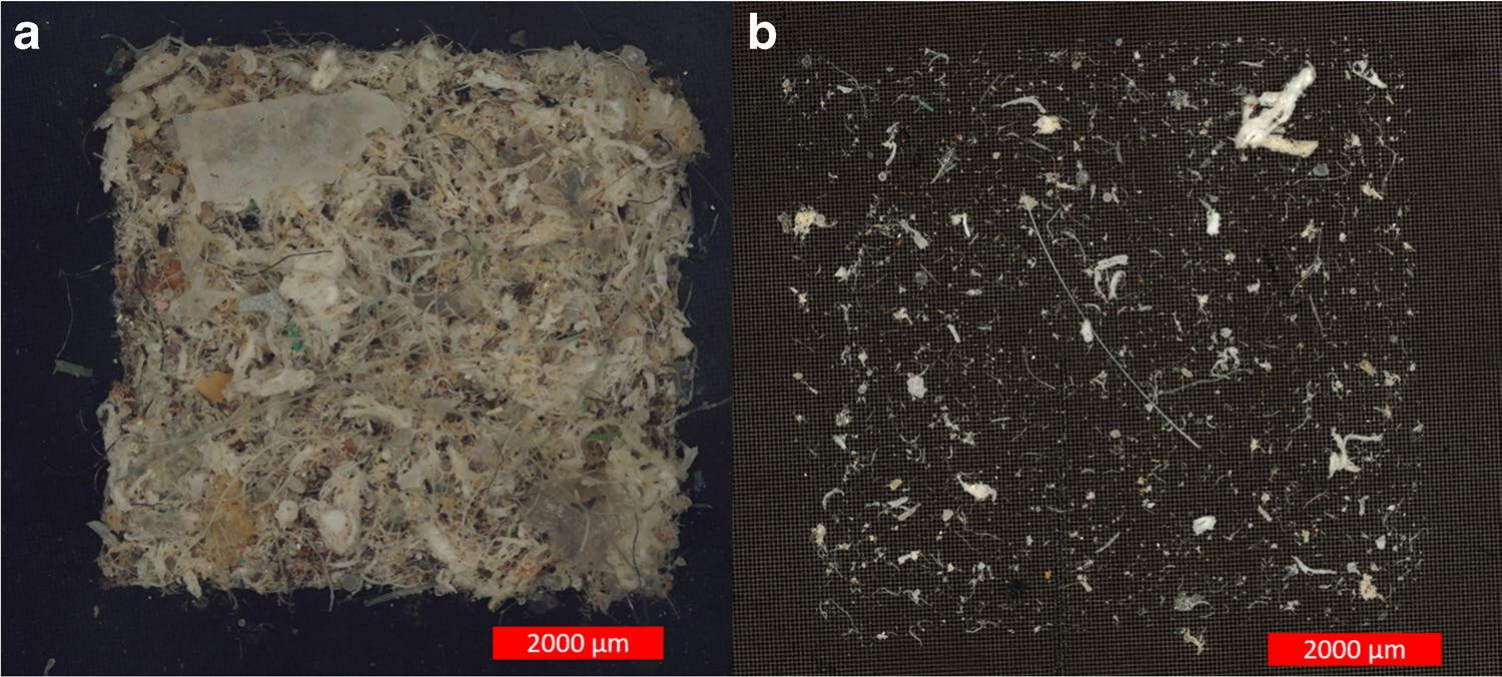
A PP recovery sample after sample purification (**a**) and an aliquot of the same sample (**b**)

Even though the water parameters deviated from those of real WWTP samples, the analysis filter was typically loaded for a wastewater sample. Beside non-target MPPs (PE and PS), many non-MP particles were present on the filter. Subsampling was necessary. Large particles, fibers, and agglomerates made the analysis by particle recognition difficult, but realistic (see Fig. 6). Further research should investigate the recovery rate of the sampling apparatus (hoses, filter, pump), comparable to Funck et al. [9]. With this information, it would be possible to distinguish between the particle losses in the sample apparatus and the losses due to sampling conditions. Optimization measures of the sampling device (see section “Solution approaches to increase MP recovery”) could be validated with such experiments. A microplastic suspension should be dosed as directly as possible into the device to avoid losses in, e.g., storage tanks.

### PVC recovery rate

The recovery rate for the PVC-MPPs is adequate (78 ± 14%). It showed that MPPs with a density higher than water can be sampled from the middle of a water column of a technical and turbulent mixed stream. The recovery rate is higher than the average recovery rate reported by Dimante-Deimantovica et al. [1] for an MP analysis protocol with seven transfer steps and comparable particle size (40%, 100 µm). The analysis protocol in this study had eight transfer steps. The results of this study are higher than the results obtained by Bordós et al. [8] for PVC-MPPs without sample preparation (100–300 µm, 1.8–24.6%). However, the PVC-MPPs used were nearly round and shaped homogenously. Usually, secondary MPPs are shaped very heterogeneously, which may influence the recovery rate. In addition, other factors such as aging processes or the formation of biofilms on the MPPs’ surface can have an effect. Regarding methodological differences, the results of recovery rates of spectroscopic analyses are not comparable to thermoanalytical ones.

The decrease in the average size of the PVC-MPPs in the recovery rate and the increase in the recovery rate with decreasing diameter do not correspond with the results of other studies and with the PP results in this study. Usually, the recovery rate decreases with decreasing MPP size. There are two reasonable explanations for this deviation: (a) the PVC-MPPs fragmented due to the oxidative sample treatment with H_2_O_2_ and NaClO. Wolff et al. [23] showed that PVC exhibits resistance to the sample purification procedure. This result corresponds with Bürkle [28] and Ehrenstein and Pongratz [29], who described PVC’s high to limited resistance, depending on material properties. However, these resistance tests have not been carried out with MP. It can be assumed that the large surface area to the total size of the particles can have a negative effect on the resistance. (b) Despite the turbulent conditions in the experimental flume, a higher percentage of the larger MPPs sedimented. The sedimentation velocity is proportional to the square of the particle diameter (Stokes equation).

### PP recovery rate

The recovery rate for the PP-MPPs is adequate for the size classes 50–100 µm (59%) and 100–140 µm (61%) but inadequate for 20–50 µm (9%). However, this low result is not surprising, since other studies have reported a large decrease in the recovery rate with decreasing particle size, even in less complex matrices such as tap water (e.g., [2]). Bordós et al. [9] reported an even lower recovery rate of 0.9–9.7% for large PP-MPPs (1000–1500 µm). Due to the realistic particle morphology and the concentration of MPPs in this study, it can be assumed that the results are comparatively realistic. However, just as for the PVC-MPPs, the PP-MPPs were not covered with a biofilm or embedded in activated sludge flocs, which may have influenced the results, especially during sampling. In future studies, MPPs should be covered with a biofilm as described by Bannick et al. [10]. One possibility would be to precondition the particles in a fine-mesh net in a WWTP aeration tank for several days. However, biofilm growth poses new challenges for particle dosing.

Oxidation of the PP particles through H_2_O_2_ and NaClO, resulting in low recovery rates, is unlikely. As for PVC, Wolff et al. [23] also proved a good resistance of PP to the applied methods.

While the PVC particles may have sedimented in the flume (*ρ* = 1400 kg/m^3^, see section  “PVC recovery rate”), the PP particles (*ρ* = 900–910 kg/m^3^) could have floated despite the turbulent conditions. However, since small particles float more slowly than larger ones, this is not likely, as the recovery rate for this fraction is the lowest.

Due to the low recovery rate, the PP concentrations determined were just slightly higher than the flume blank value. Although the values could be statistically distinguished from each other, higher concentrations should be used for future experiments to increase analytical confidence. The fact that concentrations in the effluent of wastewater treatment plants may be higher than previously assumed (see section “Conclusion”) also reinforces this.

### Solution approaches to increase MP recovery

There are several ways to increase MP recovery: sampling should be performed under isokinetic conditions. For this purpose, a pump controlled by the sampling flow rate should be used. To minimize the influence of the blocking of the cartridge filters on the sampling volume flow, the filter should be placed at the pressure side of the pump. Since pumps are difficult to clean and polymeric pump components may contaminate a sample, this may lead to contamination control problems. Another way to reduce blocking could be the use of a filter cascade. However, since each filter of a cascade needs to be treated in the sample preparation process, the number of transfer steps increases with more filters. This may lead to higher particle losses during sample preparation. A possibility to solve the influences of blocking filters on the sampling volume flow and the kinetic conditions could be the use of a buffer tank. To mitigate flotation, sedimentation, and adherence of MPPs, the tank should be stirred. The volume of the tank should not be too large since it needs to be made of stainless steel (transport) and should be easy to clean. The content of a buffer tank must be sampled (filtered) in total and cannot be rinsed (contamination control). Other filter membrane materials should be tested for sampling and sample transfer filters. The membrane must provide the lowest possible depth filtration. Beside optimization of the sampling, the sample preparation should be improved by reducing transfer steps where possible, e.g., by reducing the number of steps of oxidative treatment.

## Conclusion

The results suggest that emissions from WWTPs may be underestimated, especially for the small MPP size fractions < 50 µm, which are usually predominant in real samples [17, 24–27]. If the recovery rate determined in this study applies to real samples, this would mean that most MPPs < 50 µm are not detected, even if they are the MPPs most frequently found.

Further research on this topic is necessary. The determination of the error in analysis protocols is very important to a further homogenization and standardization of MP analysis. Besides, without reliable data on WWTP emissions, the implementation of appropriate technical reduction measures will be difficult. The accurate determination of small MPPs is very important to the ecotoxicological risk assessment of MP as well, as smaller MPPs are suspected to be more dangerous [11].

Lastly, research activities to detect microplastics in complex environmental matrices such as wastewater and surface waters should be intensified to develop and validate robust detection methods for microplastics ≥ 10 µm.
